# Emergence Angle and Emergence Profile in Implant-Supported Restorations: A Scoping Review

**DOI:** 10.3390/dj14040236

**Published:** 2026-04-15

**Authors:** Vladimir Prpic, Sven Gojsovic, Petar Kosec, Stanko Skec, Amir Catic

**Affiliations:** 1 Department of Fixed Prosthodontics, School of Dental Medicine, University of Zagreb, Gunduliceva 5, 10000 Zagreb, Croatia; sgojsovic@sfzg.unizg.hr; 2 Chair of Design, Faculty of Mechanical Engineering and Naval Architecture, University of Zagreb, Ivana Lucica 5, 10000 Zagreb, Croatia; petar.kosec@fsb.unizg.hr (P.K.); stanko.skec@fsb.unizg.hr (S.S.); 3 Department of Fixed Prosthodontics, School of Dental Medicine, University of Zagreb, Clinical Hospital Centre Zagreb, Gunduliceva 5, 10000 Zagreb, Croatia; catic@sfzg.unizg.hr

**Keywords:** emergence angle, emergence profile, implant-supported restoration, computer-aided design, prosthodontics

## Abstract

**Background/Objectives**: Prosthodontic restoration design plays a key role in the long-term success of implant-supported treatments and in maintaining peri-implant tissue health. Inadequate emergence angles and profiles can compromise tissue stability and negatively influence clinical outcomes. Generative design, as an algorithm-driven optimization approach, requires the definition of key parameters in advance to guide the process and determine the final shape of the hybrid implant abutment. **Methods**: A detailed literature review of the PubMed and Scopus databases was performed to find appropriate studies published up to 1 December 2025. Studies that investigated the emergence angle and emergence profile of implant-supported restorations were included. Seventeen studies fulfilled criteria and were included in the final analysis. **Results**: While the optimal emergence angle is still debatable, the literature suggests that an angle less than 30° may be beneficial. However, a concave emergence profile of implant-supported restoration has a significant role in improving stability and maintaining peri-implant health. **Conclusions**: Careful characterization and evaluation of the included parameters provide useful insights for generative design workflows, enabling the creation of implant abutment designs that maintain a balance between mechanical performance and biological compatibility.

## 1. Introduction

Implant abutments serve as the connecting components between the implant body and the implant-supported restorations. These components are typically made from titanium or all-ceramic materials and serve as transgingival structures that stay in constant contact with the peri-implant soft tissue. More specifically, implant abutments are positioned in a critical, bacteria-rich zone between the peri-implant bone and the oral cavity [1,2]. Selection and design of an adequate implant abutment is critical for attaining efficiency, aesthetic excellence, and long-term stability in implant prosthodontic therapy [3,4]. The most common types of implant abutments are prefabricated, customized, and two-piece structures made of biomaterials and designed to support cement-retained or screw-retained prosthodontic restorations [5]. Titanium is a well-established implant abutment material with high biological compatibility and mechanical strength, making it appropriate for the majority of clinical applications [6]. Nonetheless, the metallic hue of titanium has aesthetic limitations, particularly in the anterior region. As a result, ceramic implant abutments were offered as an alternative, with zirconia becoming the favored material due to its advanced mechanical properties. In addition, biocompatibility of zirconia is comparable to that of titanium [3,5,7].

With the rapid development of CAD/CAM (Computer-Aided Design/Computer-Aided Manufacturing) technologies, design and production of implant abutments has increasingly moved towards digitally driven workflows. This approach reduces the need for manual steps (impression taking, model preparation and manual shaping of implant abutment), simplifies the fabrication process and improves accuracy and reproducibility. Moreover, CAD/CAM-based implant abutments can be personalized to meet specific clinical situations [3]. A more recent innovation in dental implantology is the hybrid implant abutment concept. Hybrid implant abutments combine the best qualities of zirconia (esthetics and biocompatibility) and titanium (mechanical performance). Importantly, hybrid implant abutments do not have unfavorable impact on the implant-abutment interface [3,5,8].

Peri-implant mucositis is described as a reversible inflammatory response confined to the soft tissues surrounding a dental implant. It shows clinical and pathological similarities to gingivitis, as it does not involve loss of the alveolar bone. Conversely, peri-implantitis is defined as a progressive, nonreversible inflammatory condition that affects both the peri-implant soft tissue complex and underlying alveolar bone, ultimately leading to loss of osseointegration and potential implant failure [9,10,11]. Compared with natural teeth, peri-implant soft tissues demonstrate a diminished ability to form effective epithelial and connective tissue seals [12]. The biological integrity of peri-implant tissues is strongly influenced by the shape and design of the implant–abutment–restoration system, particularly under exposure to persistent oral microbial biofilm [13]. Prosthodontic characteristics such as emergence angle and emergence profile have potential to influence short-term clinical performance and long-term health of surrounding hard and soft tissues [13].

The emergence profile represents the part of the implant–abutment–restoration system that extends from the alveolar bone to the free gingival margin. The subgingival contour of the implant-supported restoration’s emergence profile can be divided into three zones: E (esthetic) zone, B (bounded) zone, and C (crestal) zone [14,15]. The E zone is defined as subgingival area located 1 mm below the free gingival margin. It should resemble closely the shape of a natural crown by replicating the features of the extracted tooth or its contralateral counterpart. The B zone refers to the area located apical to the E zone. This zone, which measures 1–2 mm, is determined by the soft tissue thickness and the position of a dental implant. Finally, the C zone is the area nearest to the implant platform and usually measures 1–1.5 mm. To achieve optimal aesthetic results, it is essential to minimize pressure on the surrounding hard tissues [14,15]. The EBC emergence profile zones serve as a guide for designing the emergence profile, enabling optimal esthetic outcomes while maintaining healthy, stable tissues and minimizing the possibility of alveolar bone loss and soft tissue recession. Emergence profile contours are typically classified as convex, concave, or straight [16]. Convex emergence profiles provide enhanced mechanical support to peri-implant soft tissues and are useful in areas where additional tissue volume is required. However, if this design is not precisely tailored to the patient’s anatomical characteristics, it may promote biofilm retention and compromise soft tissue accommodation [13,17]. Concave emergence profiles gradually narrow from the implant platform toward the restoration, creating additional space that allows soft tissue expansion and supports improved vascularization. This design is particularly useful in the anterior zone, as it creates a smooth, natural-looking transition and minimizes the risk of marginal bone changes [17,18]. Straight emergence profiles have a neutral, linear shape from the implant platform to the restoration. These designs are most frequently used in distal regions, where aesthetic demands are lower and greater emphasis is placed on functional reliability and ease of cleaning, making straight design highly effective [13,17]. As a result, a well-designed emergence profile of an implant-supported restoration reduces pressure on the adjacent tissues, enhances soft tissue integration, and lowers the probability of inflammation. Furthermore, gradual and precisely structured transitions in the emerging profile increase peri-implant tissue sealing and prevent microbial adhesion, both of which are critical for alveolar bone maintenance [17]. In addition, the emergence angle is specified as the angle formed between the average tangent of the transitional contour and the implant body’s long axis [16,19]. An excessively wide emergence angle of implant abutments may be linked to early marginal bone loss [20].

Parametric models enable adjustments to the dimensions and shape of a configuration (e.g., hybrid implant abutment), acting as foundational templates while keeping the core’s integrity [21]. However, manually modifying complex parametric designs can be challenging, time-consuming, and error-prone. Consequently, the need for more efficient design methods becomes evident [21]. From this perspective, computer-aided design leverages iterative algorithms to explore, optimize, and refine a wide range of design possibilities, building on the flexibility offered by parametric modeling [22]. Generative design enables the efficient creation of highly personalized solutions that meet specific biological and mechanical requirements, using computational power to explore complex, multidimensional designs in a scalable and effective manner [21,23]. In implant prosthodontics, generative design enables the exploration of numerous design alternatives to identify the optimal configuration for structures like hybrid implant abutments [3].

Current evidence on standardized criteria for the optimal structural design of hybrid implant abutments is limited, and no universally accepted guidelines exist to guide evidence-based clinical decisions. A recent study on the application of generative design in implant prosthodontics highlighted the important role of transmucosal and prosthodontic abutment heights in achieving predictable biological integration and optimizing mechanical stability [3]. Accordingly, clinical outcomes for peri-implant soft and hard tissues are strongly influenced by careful adjustment of implant design parameters, such as the emergence angle and profile of implant-supported restorations. These parameters play a crucial role in guiding the development and maturation of adjacent implant tissues. However, creating an initial parametric model for the generative design of hybrid implant abutments involves systematically identifying and integrating additional design variables found in the available literature.

Platform-switching, which involves using a narrower abutment on a wider implant platform, helps preserve crestal bone and reduces marginal bone loss, improving long-term implant stability. The type of implant–abutment connection—whether internal or external—affects mechanical stability, stress distribution, and microgap formation, influencing both biological outcomes and prosthodontic longevity. Esthetic sites, such as the anterior maxilla, demand careful management of soft tissue and bone to achieve natural appearance, whereas posterior sites prioritize functional load bearing over appearance. Platform-switching and stable connections are especially critical in esthetic regions to prevent recession and maintain papillae. Overall, understanding these factors allows clinicians to optimize both functional and esthetic outcomes for implant-supported restorations. The variables considered—namely platform switching, the type of implant–abutment connection, and the differentiation between esthetic and posterior sites—were not included within the primary scope of this review, as these factors have already been comprehensively examined in the existing body of literature. Addressing these factors would necessitate a more comprehensive study design, which exceeds the objectives of the present investigation.

The purpose of the study was to conduct a review of the scientific evidence on the emergence angles and profiles of implant-supported restorations in order to determine ideal values, morphological characteristics, and the impact on surrounding tissues. The main objective of this study was to outline the essential preparatory processes necessary for the successful application of generative design in implant prosthodontics.

## 2. Materials and Methods

**Figure 1:**
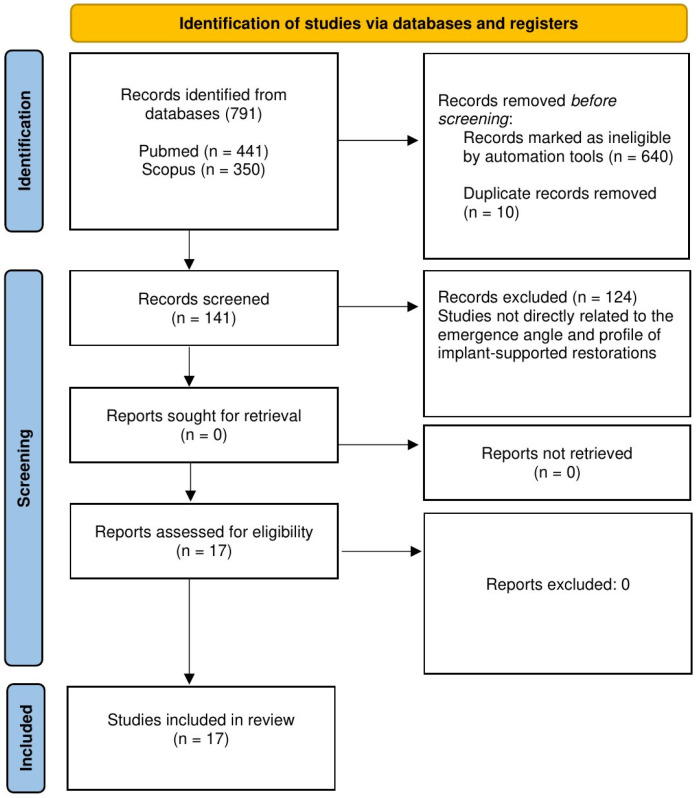
Flowchart based on PRISMA guidelines illustrating the process of record identification, screening, and selection during the search.

The present study was registered in the Open Science Framework (OSF) public registry (Registration DOI: 10.17605/OSF.IO/Q7VDS) and reported according to the PRISMA Extension for Scoping Reviews (PRISMA-ScR) guidelines [24]. A literature review was carried out to identify studies relevant to the objectives of the present scoping review. Studies published in the past five years that focused on the emergence angle and emergence profile of implant-supported restorations were considered for inclusion. The assessment of risk of bias was not conducted, as this study is classified as a scoping review. A five-year publication window was selected to capture the most recent studies and to ensure the inclusion of the most current and reliable evidence in the review. This timeframe enables the analysis to reflect the most recent advances and emerging trends within the field. Studies have to be full text, published in English, and involve human subjects in order to meet additional inclusion requirements. Studies were excluded if they consisted of case series, editorials, or interview articles. A systematic search of the PubMed and Scopus databases was conducted to find eligible studies published up to 1 December 2025. To identify studies investigating the emergence angle of implant-supported restorations, the keyword “emergence angle of implant restoration” was employed. Similarly, for the identification of studies examining the emergence profile of implant-supported restorations, the keyword “emergence profile of implant restoration” was utilized. The detailed PRISMA checklist is provided in Table S1 in the Supplementary Materials [24]. A PRISMA-style flow diagram (Figure 1) illustrates the ultimate number of studies included in the scoping review. Two experienced reviewers (V.P. and S.G.) first screened the titles of all collected records based on predefined inclusion criteria. Abstracts of potentially relevant studies were then assessed, followed by full-text evaluations for studies that met the eligibility criteria. Final inclusion in the analysis was determined by agreement between the primary reviewers. In cases of disagreement or uncertainty, two supplementary reviewers (S.S. and A.C.) were consulted to reach agreement. Data extraction included the following parameters: authors, year of publication, study title, study type, sample size, and study outcomes (Table 1 and Table 2).

## 3. Results

In total, 791 studies were initially retrieved from the literature search. Before the screening procedure, 650 records were removed: 640 by automation tools and 10 as duplicates. A total of 141 records were included in the screening procedure, of which 124 were excluded for not meeting the inclusion criteria. Ultimately, 17 studies fulfilled the requirements and were selected in the final analysis (Figure 1).

Figure 1 presents a PRISMA flowchart illustrating the process of record identification, screening, and selection during the literature search. Ten studies [5,17,25,26,27,28,29,30,31,32] dealing with the emergence angle of implant-supported restoration were included in the present scoping review. Given studies investigated influence of the emergence angle of implant-supported restoration on peri-implant tissue health. Five studies [5,25,26,27,32] suggest that maintaining an emergence angle under 30° contributes to minimizing marginal bone loss and lower the risk of peri-implant inflammation. Neckel et al. [5] conducted a cross-sectional study on 462 implants and reported that the combination of an emergence angle of less than 30° and a concave emergence profile was associated with a significant reduction in annual peri-implant bone loss. Lin et al. [25] performed a systematic review and meta-analysis, demonstrating that emergence angles below 30°, in combination with a concave or straight emergence profile, were associated with a reduced risk of peri-implantitis. Misch et al. [26] carried out a retrospective study on 192 implants and found that an abutment height greater than 2 mm plays a significant role in reducing the incidence of peri-implantitis and marginal bone loss associated with restoration emergence angles ≥30° around bone-level implants. Majzoub et al. [27] conducted a retrospective study on 83 implants and demonstrated that restoration emergence angles greater than 30° were significantly associated with early-stage (up to 2 years) peri-implant marginal bone loss. Finally, a digital comparative study by Yu et al. [32], including 100 implants, showed that cantilever implant-supported restorations for first molar replacements exhibit more favorable emergence angles compared to non-cantilever designs, with a significantly lower proportion exceeding the clinically relevant 30° threshold. Other four studies (one prospective cohort and three retrospective) [28,29,30,31] did not demonstrate evident correlation between the emergence angle and peri-implant soft-tissue health or marginal bone loss, while one comprehensive review [17] stated that there is still no consensus on the relationship between emergence angle and marginal bone loss. A consolidated overview of the extracted data from the reviewed studies is presented in Table 1.

Seven studies [5,14,16,17,25,33,34] focused on evaluating the influence of the emergence profile of implant-supported restorations on peri-implant health. Four studies [5,25,33,34] precise that concave emergence profile is associated with reduced marginal bone loss and increased stability of the soft tissue margin. Neckel et al. [5] carried out a cross-sectional study and demonstrated that the combination of an emergence angle of less than 30° and a concave emergence profile was associated with a significant reduction in annual peri-implant bone loss. Lin et al. [25] performed a systematic review and meta-analysis and reported that concave or straight emergence profiles were associated with reduced marginal bone loss. Siegenthaler et al. [33], in a randomized controlled clinical trial involving 47 implants, demonstrated that the use of implant-supported provisional restorations with a concave emergence profile resulted in greater mucosal margin stability compared with convex profiles up to 1 year post-loading. Endres et al. [34], in a randomized controlled trial comprising 47 implants, reported that a concave emergence profile conferred superior soft tissue stability, particularly in preventing mid-facial recessions. The remaining three studies (review, narrative review, and comprehensive review) [14,16,17] emphasized the importance of carefully considering the emergence profile due to its impact on peri-implant health. Table 2 summarizes the studies that assessed the emergence profile of implant-supported restorations.

## 4. Discussion

A thorough parametrization procedure, which identifies and quantifies biomechanical and anatomical factors, is essential before applying generative design to hybrid implant abutments. Generative design algorithms can function under realistic clinical conditions, with parametric models serving as foundational templates that simplify customization. They enable designers to modify dimensions, shapes, and material properties while maintaining the integrity of the core structure [22]. Without this step, generative designs may not fulfill the practical and biomechanical requirements of dental rehabilitation. Accordingly, this study was conducted to review and synthesize the evidence on the emergence angle and profile of implant-supported restorations.

### 4.1. Studies Dealing with Emergence Angle of Implant-Supported Restorations

Ten studies [5,17,25,26,27,28,29,30,31,32] that investigated the emergence angle of implant-supported restorations reported the following findings. Neckel et al. [5] examined two types of implant abutments—prefabricated titanium abutments and customized hybrid zirconia abutments in fixed dental prosthesis. The study found that an emergence angle < 30°, together with a concave emergence profile, reduced peri-implant bone loss, and this effect did not depend on the type of implant abutment [5]. These outcomes suggest that long-term peri-implant stability relies not only on material choice but, more critically, on maintaining a minimal emergence angle and establishing a concave emergence profile [5]. These observations are consistent with the study by Lin et al. [25], who reported that emergence angles < 30° and a concave or straight emergence profile were correlated with a reduced risk of peri-implantitis. Misch et al. [26] analyzed implant radiographs obtained 12–18 months after crown placement (T0) and one year afterwards (T1). The emergence angle was evaluated on both the mesial and distal sides using radiographs. Authors measured an angle between the long axis of a implant and the shape of the restoration as it emerged from the implant platform. The study found that the biological risk linked to a wide (>30°) emergence angle becomes clinically significant only when the transmucosal height of the implant abutment is under 2 mm. Furthermore, Majzoub et al. [27] investigated the influence of the emergence angle of a restoration on marginal bone loss in implants affected by peri-implantitis. Implants affected by peri-implantitis and with follow-up at 1 year (T1) and 2 years (T2) were included in the analysis. Additionally, each case required full documentation completed within six months before the diagnosis (Tb), with no detectable signs of peri-implantitis. Marginal bone level changes were assessed for the intervals Tb-T1 and T1–T2 [27]. Substantial marginal bone loss was observed during the early period following peri-implantitis diagnosis, and this progression was associated with the emergence angle (>30°) of implant-supported restoration [27]. Yu et al. [32] investigated the emergence angle of cantilever versus non-cantilever digital designs in implant-supported restorations. A total of 100 patients who were missing their first molar underwent both digital impression and cone beam computed tomography (CBCT) imaging. Cantilever and non-cantilever designs were created digitally for every patient [32]. This study reported significant differences in the emergence profiles of implant-supported restorations with cantilever extensions compared to those without [32]. Linear regression models were used to examine the relationships between emergence angles and clinical parameters. Cantilever implant-supported restorations generally exhibited smaller emergence angles than non-cantilever designs, which may contribute to improved peri-implant health. This modification enhances hygiene by making plaque easier to manage, potentially reducing the potential of peri-implant inflammation. Nevertheless, cantilevered restorations can create areas prone to food impaction and hinder interdental hygiene, highlighting the need for comprehensive patient education [32]. Generally, cantilever designs may serve as a favorable strategy in implant prosthodontics.

On the other side, Izzetti et al. [17] reviewed the literature and showed that the relationship between emergence angle and marginal bone loss still remains a topic of ongoing debate. Moreover, Tang et al. [28] evaluated influence of emergence angle (36.7 ± 13.7°) of 57 implant-supported restorations on the buccal mucosa recession and vertical bone loss. Within this range, authors did not find any meaningful link between the emergence angle and the amount of buccal vertical bone loss [28]. However, differences between study outcomes could be related to the measurement approach, since the emergence angle was often assessed using periapical radiographs without considering its relationship to the gingival margin [28]. Maghsoudi et al. [29] analyzed peri-implant care in patients receiving implant-supported fixed prostheses. The baseline evaluation was conducted approximately 8 weeks after the placement of the definitive restoration to clinically and radiographically assess the peri-implant status. Recorded parameters included peri-implant bleeding on probing, peri-implant probing depth and gingival recessions [29]. At follow-up, the emergence angle was not linked to an increase in peri-implant probing pocket depth and no differences were observed between angles below 30° and those of 30° or more [29]. Authors stated that the effect of the emergence angle may be less evident in patients who are well maintained or in cases where other risk factors are effectively managed [29]. In addition, Lops et al. [30,31] conducted two studies examining how prosthodontic emergence angles affect peri-implant soft tissue health and the maintenance of marginal bone. Peri-implant soft tissue stability seems to remain unaffected by the value of the emergence angle, as long as an appropriate (concave) emergence profile is achieved for anterior implant-supported restorations—even when the angle exceeds 30° [30]. Likewise, when the implant-abutment interface is mechanically strong and stable, an emergence angle between 30° and 50° is unlikely to affect the preservation or stability of marginal bone levels [31]. The same applies to emergence angles less than 30° [31]. Overall, these findings suggest that well-designed emergence profiles and stable implant-abutment connections can help reduce the potential biological effects of larger emergence angles within this range [30,31]. The included studies indicate that understanding the relationship between the emergence angle of implant-supported restorations and peri-implantitis can provide valuable guidance for dental professionals and technicians to optimize treatment outcomes. Emergence angles should promote healthy soft tissue adaptation, maintain a stable mucosal seal, and minimize plaque accumulation, thereby facilitating oral hygiene and long-term care. Although an angle <30° might be seen as a potentially favorable design goal, the evidence is limited, and additional research is needed.

### 4.2. Studies Dealing with Emergence Profile of Implant-Supported Restorations

Seven studies [5,14,16,17,25,33,34] examining the emergence profile of implant-supported restorations reported the following information. The study by Neckel et al. [5] examined various combinations of emergence angles and profiles, finding that emergence angle under 30° and a concave shape were associated with significantly less bone loss compared to those with an angle over 30° and a convex profile. Assessment of multiple combinations of emergence angle and emergence profile suggests that the shape becomes particularly important when the emergence angle exceeds 30°, while no significant effect is seen for angles smaller than 30° [5]. Gervyte et al. [14] conducted a review that stated that implant position and soft tissue condition must be carefully assessed to achieve an optimal esthetic result. In conclusion, a clear understanding of the distinctive characteristics of the emergence profile is pivotal for achieving the best possible esthetic outcome [14]. Hamilton et al. [16] performed a review to assess the available evidence regarding how implant position, implant design and prosthodontic design influence the risk to peri-implant tissue health and concluded that prosthodontic design of an implant-supported restoration is closely associated with the long-term condition and maintenance of peri-implant tissue health. One of the key factors in achieving a successful implant restoration is placing the implant in the ideal position. Inadequately positioned implants frequently result in a restorative emerging profile at the implant-abutment interface, which can make it challenging for patients to maintain optimal oral hygiene [16]. Additionally, Izzetti et al. [17] carried out a comprehensive review to assess how peri-implant marginal bone stability is impacted by the emergence profile of implant-supported restorations, highlighting its possible function in promoting long-term peri-implant tissue health. The authors highlighted that careful management of the emergence profile is particularly important for implant-supported restorations in patients with a thin soft tissue phenotype [17]. Lin et al. [25] conducted a review examining how various prosthodontic design parameters, including emergence angle and profile, affect peri-implant bone resorption. Their findings showed that concave or straight emergence profiles were linked to a lower risk of peri-implantitis [25]. Comparable evidence was reported by Siegenthaler et al. [33], who investigated how the emergence profile (convex and concave) of implant-supported restorations affects the stability of the peri-implant mucosal margin during the 12 months period after the placement of a definitive restoration. A total of 47 patients who received an implant in the anterior maxilla were randomly assigned to one of three groups: convex—provisional and definitive restorations had a convex emergence profile; concave—provisional and final restorations had a concave profile; and control—no provisional restoration was used, with only a healing abutment followed by the definitive restoration. Clinical evaluations were performed at baseline, 6 months, and 12 months for all participants. The study found that implant-supported provisional restorations with a concave emergence profile promote greater stability of the peri-implant mucosal margin compared with convex profiles [33]. Finally, Endres et al. [34] assessed the three-year clinical and radiographic outcomes of implant-supported restorations with either convex or concave emergence profiles. A total of 47 patients received a single implant in the aesthetic zone. In convex and concave groups, the definitive crowns were designed to replicate the emergence profiles of the provisional restorations. Follow-up were conducted at baseline, 6 months, 1 year, and 3 years. The shape of the emergence profile plays an important role in maintaining soft tissue stability, especially during the first year after placement of the definitive crown, when most changes occur. A concave profile may encourage coronal soft tissue creeping by creating extra space, allowing the tissue to adapt more naturally. Consequently, a concave emergence profile is recommended in the aesthetic zone, as it helps preserve the mid-facial mucosal margin and lowers the risk of recession [34]. The emergence profile of implant-supported restorations also affects soft tissue adaptation, vascularization, and aesthetic outcomes, with concave profiles generally promoting greater tissue thickness and improved stability. Convex emergence profiles in implant-supported restorations were generally associated with larger emergence angles, whereas straight or concave profiles tended to have smaller angles. Therefore, careful optimization of both emergence angle and profile is essential for ensuring long-term functional, hygienic, and aesthetic success.

### 4.3. Limitations

This study has three limitations. First, the selected studies were heterogeneous, varying in design, methods, and outcomes, which could affect the generalizability of the findings. Second, there is a possibility of missing data, as only studies published in English and indexed in two databases were considered. Finally, the absence of a risk of bias analysis, although not required in a scoping review, may be acknowledged as a third limitation of the study. These factors should be taken into account when interpreting the findings.

## 5. Conclusions

A notable proportion of studies indicate that an emergence angle < 30° may be beneficial in reducing marginal bone loss. However, this trend is not consistently supported across all included data. Although an angle < 30° may be seen as a potentially favorable design target, the evidence is still inconclusive and needs to be confirmed through randomized controlled trials. Included studies suggest that concave emergence profile is associated with soft tissue stability and reduced marginal bone loss. These findings (emergence angle below 30° and a concave emergence profile) provide evidence-based guidance for designing hybrid implant abutments and offer a strong foundation for creating parametric models in generative design.

## 6. Future Directions

Future investigations into the emergence profile and emergence angle of implant-supported restorations are expected to increasingly focus on personalized design approaches. The implementation of generative design in implant prosthodontics is still under active development, with standardized technical methodologies and clinically established protocols yet to be realized. The application of generative design to the customization of hybrid implant abutments may substantially influence treatment planning by enabling the development of designs that enhance biological compatibility while preserving functional and prosthodontic performance.

## Figures and Tables

**Table 1:** Studies focused on the emergence angle of implant-supported restorations.

Authors, Year	Title	Study Type	Sample Size	Outcome
Neckel et al. (2024) [5]	Cross-sectional analysis comparing prefabricated titanium to individualized hybrid zirconia abutments for cemented zirconia based fixed dental prostheses: a critical concept assessment	Cross-sectional study	102 patients 462 implants	The combination of an emergence angle of <30° and a concave emergence profile significantly improved the annual peri-implant bone loss
Izzetti et al. (2025) [17]	Influence of Prosthetic Emergence Profile on Peri-Implant Marginal Bone Stability: A Comprehensive Review	Comprehensive Review	/	The relationship between emergence angle and marginal bone loss is still debated in the literature
Lin et al. (2025) [25]	The influence of prosthetic designs on peri-implant bone loss: An AO/AAP systematic review and meta-analysis	Systematic review and meta-analysis	/	Emergence angles < 30° and a concave/straight emergence profile were linked to decreased peri-implantitis risk
Misch et al. (2025) [26]	Combined Effect of Abutment Height and Restoration Emergence Angle on Peri-Implant Bone Loss Progression: A Retrospective Analysis	Retrospective study	119 patients 192 implants	Abutment height greater than 2 mm plays a significant role in reducing the experience of peri-implantitis and marginal bone loss related to ≥30° restoration emergence angle around bone-level implants
Majzoub et al. (2021) [27]	Influence of restorative design on the progression of peri-implant bone loss: A retrospective study	Retrospective study	65 patients 83 implants	Restoration emergence angles of >30° were found to be significantly associated with early stage (up to 2 years) of peri-implant marginal bone loss in peri-implantitis
Tang et al. (2025) [28]	Influence of buccal mucosa width/height ratio, emergence profile and buccal bone width on peri-implant tissues: a prospective one-year study	Prospective cohort study	52 patients 57 implants	The emergence angle of the restorations in the present study was 36.7 ± 13.7°. There was no correlation between the emergence angle and buccal vertical bone loss in this range
Maghsoudi et al. (2025) [29]	Retrospective Evaluation of Peri-Implant Maintenance in Patients With Implant-Supported Fixed Prostheses	Retrospective study	68 patients 108 implants	The emergence angle was found not to be a predictor of increased peri-implant probing pocket depth at follow-up and neither any differences were found between the <30° and ≥30° groups
Lops et al. (2022) [30]	Association between Peri-Implant Soft Tissue Health and Different Prosthetic Emergence Angles in Esthetic Areas: Digital Evaluation after 3 Years’ Function	Retrospective study	57 patients 220 implants	Peri-implant soft-tissue health does not seem to be influenced by emergence angle itself when a proper emergence profile is provided for implant-supported reconstructions in anterior area
Lops et al. (2022) [31]	Marginal Bone Maintenance and Different Prosthetic Emergence Angles: A 3-Year Retrospective Study	Retrospective study	74 patients 312 implants	The marginal bone loss change does not seem to be influenced by the emergence angle for implants with a stable internal conical connection and platform-switching of the abutment diameter
Yu et al. (2025) [32]	Emergence angle and profile at implant-supported crowns with and without cantilever extension: A digital study in molar sites	Digital comparative study	100 patients 100 implants	This digital comparative study demonstrates that cantilever implant-supported restorations for first molar replacements exhibit more favorable emergence angles than non-cantilever designs, with significantly fewer cantilever profiles exceeding the clinically significant 30° threshold

**Table 2:** Studies focused on the emergence profile of implant-supported restorations.

Authors, Year	Title	Study Type	Sample Size	Outcome
Neckel et al. (2024) [5]	Cross-sectional analysis comparing prefabricated titanium to individualized hybrid zirconia abutments for cemented zirconia based fixed dental prostheses: a critical concept assessment	Cross-sectional study	102 patients 462 implants	The combination of an emergence angle of <30° and a concave emergence profile significantly improved the annual peri-implant bone loss
Gervyte et al. (2023) [14]	Emergence profile management in the esthetic zone	Review	/	For the most favorable esthetic outcomes in the anterior zone all important factors, especially hard and soft tissue presence and conditions, implant position, and optimal emergence profile should be carefully considered for every individual clinical situation
Hamilton et al. (2023) [16]	Implant prosthodontic design as a predisposing or precipitating factor for peri-implant disease: A review	Narrative review	/	The prosthetic design of an implant restoration has a close relationship to the future of peri-implant health
Izzetti et al. (2025) [17]	Influence of Prosthetic Emergence Profile on Peri-Implant Marginal Bone Stability: A Comprehensive Review	Comprehensive Review	/	It appears to be of utmost importance to correctly manage the emergence profile of the implant-prosthetic restoration, especially in cases characterized by a thin phenotype
Lin et al. (2025) [25]	The influence of prosthetic designs on peri-implant bone loss: An AO/AAP systematic review and meta-analysis	Systematic review and meta-analysis	/	Concave/straight emergence profile was found to be associated with reduced marginal bone loss
Siegenthaler et al. (2022) [33]	Anterior implant restorations with a convex emergence profile increase the frequency of recession: 12-month results of a randomized controlled clinical trial	Randomized controlled clinical trial	47 patients 47 implants	The use of implant-supported provisionals with a concave emergence profile results in a greater stability of the mucosal margin compared with a convex profile up to 1 year post-loading
Endres et al. (2025) [34]	Convex Versus Concave Emergence Profile of Implant-Supported Crowns in the Aesthetic Zone: 3-Year Results of a Randomized Controlled Trial	Randomized Controlled Trial	47 patients 47 implants	A concave profile showed superior soft tissue stability, particularly in preventing mid-facial recessions

## Data Availability

The original contributions presented in this study are included in the article and Supplementary Material. Further inquiries can be directed to the corresponding author.

## Supplementary Materials

The following supporting information can be downloaded at: https://www.mdpi.com/article/10.3390/dj14040236/s1, Table S1: Preferred Reporting Items for Systematic reviews and Meta-Analyses extension for Scoping Reviews (PRISMA-ScR) Checklist.

## References

[1] Hofmann P., Kunz A., Schmidt F., Beuer F., Duddeck D. (2023). Influence of exposure of customized dental implant abutments to different cleaning procedures: An in vitro study using AI-assisted SEM/EDS analysis. Int. J. Implant. Dent..

[2] Kim S., Choi C., Cha Y., Chang J.S. (2021). The efficacy of convenient cleaning methods applicable for customized abutments: An in vitro study. BMC Oral Health.

[3] Prpic V., Kosec P., Skec S., Catic A. (2025). Influence of Design Parameters on Implant Abutment Performance: A Scoping Review. J. Funct. Biomater..

[4] Strasding M., Marchand L., Merino E., Zarauz C., Pitta J. (2024). Material and abutment selection for CAD/CAM implant-supported fixed dental prostheses in partially edentulous patients—A narrative review. Clin. Oral Implant. Res..

[5] Neckel N., Pohl J., Preissner S., Wagendorf O., Sachse C., Vach K., Heiland M., Nahles S. (2024). Cross-sectional analysis comparing prefabricated titanium to individualized hybrid zirconia abutments for cemented zirconia based fixed dental prostheses: A critical concept assessment. Int. J. Implant. Dent..

[6] Hanawa T. (2020). Zirconia versus titanium in dentistry: A review. Dent. Mater. J..

[7] Totou D., Naka O., Mehta S.B., Banerji S. (2021). Esthetic, mechanical, and biological outcomes of various implant abutments for single-tooth replacement in the anterior region: A systematic review of the literature. Int. J. Implant. Dent..

[8] Mostafavi A.S., Mojtahedi H., Javanmard A. (2021). Hybrid Implant Abutments: A Literature Review. Eur. J. Gen. Dent..

[9] Soulami S., Slot D.E., van der Weijden F. (2022). Implant-abutment emergence angle and profile in relation to peri-implantitis: A systematic review. Clin. Exp. Dent. Res..

[10] Dixon D.R., London R.M. (2019). Restorative design and associated risks for peri-implant diseases. Periodontol. 2000.

[11] Koutouzis T. (2019). Implant-abutment connection as contributing factor to peri-implant diseases. Periodontol. 2000.

[12] Dib-Zaitum I., Guadilla-González Y., Flores-Fraile J., Dib-Zakkour J., Benito-Garzón L., Montero J. (2022). Effect Morphology and Surface Treatment of the Abutments of Dental Implants on the Dimension and Health of Peri-Implant Biological Space. Materials.

[13] Mattheos N., Janda M., Acharya A., Pekarski S., Larsson C. (2021). Impact of design elements of the implant supracrestal complex (ISC) on the risk of peri-implant mucositis and peri-implantitis: A critical review. Clin. Oral Implant. Res..

[14] Gervyte J., Zidonyte Z., Trumpaite-Vanagiene R., Linkevicius T. (2023). Emergence profile management in the esthetic zone. Stomatologija.

[15] Gomez-Meda R., Esquivel J., Blatz M.B. (2021). The esthetic biological contour concept for implant restoration emergence profile design. J. Esthet. Restor. Dent..

[16] Hamilton A., Putra A., Nakapaksin P., Kamolroongwarakul P., Gallucci G.O. (2023). Implant prosthodontic design as a predisposing or precipitating factor for peri-implant disease: A review. Clin. Implant. Dent. Relat. Res..

[17] Izzetti R., Cinquini C., Nisi M., Covelli M., Alfonsi F., Barone A. (2025). Influence of Prosthetic Emergence Profile on Peri-Implant Marginal Bone Stability: A Comprehensive Review. Medicina.

[18] Cinquini C., Parisi E., Baldi N., Miccoli M., Alfonsi F., Barone A. (2025). Esthetic Outcomes of Immediately Placed Implants with Convergent Transmucosal Profiles: A Retrospective Single-Cohort Study. Int. J. Oral Maxillofac. Implant..

[19] Strauss F.J., Park J.Y., Lee J.S., Schiavon L., Smirani R., Hitz S., Chantler J.G.M., Mattheos N., Jung R., Bosshardt D. (2024). Wide Restorative Emergence Angle Increases Marginal Bone Loss and Impairs Integrity of the Junctional Epithelium of the Implant Supracrestal Complex: A Preclinical Study. J. Clin. Periodontol..

[20] Puisys A., Janda M., Auzbikaviciute V., Gallucci G.O., Mattheos N. (2023). Contour angle and peri-implant tissue height: Two interrelated features of the implant supracrestal complex. Clin. Exp. Dent. Res..

[21] Kosec P., Huic I., Martinec T., Škec S. (2025). Exploring generative design in context of mass personalization. Proc. Des. Soc..

[22] Papallo I., Martorelli M., Lamonaca F., Gloria A. (2023). Generative design and insights in strategies for the development of innovative products with tailored mechanical and/or functional properties. Acta IMEKO.

[23] Cirello A., Ingrassia T., Marannano G., Mirulla A.I., Nigrelli V., Petrucci G., Ricotta V. (2024). A New Automatic Process Based on Generative Design for CAD Modeling and Manufacturing of Customized Orthosis. Appl. Sci..

[24] Tricco A.C., Lillie E., Zarin W., O’Brien K.K., Colquhoun H., Levac D., Moher D., Peters M.D.J., Horsley T., Weeks L. (2018). PRISMA Extension for Scoping Reviews (PRISMA-ScR): Checklist and Explanation. Ann. Intern. Med..

[25] Lin G.H., Lee E., Barootchi S., Rosen P.S., Curtis D., Kan J., Wang H.L. (2025). The influence of prosthetic designs on peri-implant bone loss: An AO/AAP systematic review and meta-analysis. J. Periodontol..

[26] Misch J., Abu-Reyal S., Lohana D., Mandil O., Saleh M.H.A., Li J., Wang H.L., Ravidà A. (2025). Combined Effect of Abutment Height and Restoration Emergence Angle on Peri-Implant Bone Loss Progression: A Retrospective Analysis. Clin. Oral Implant. Res..

[27] Majzoub J., Chen Z., Saleh I., Askar H., Wang H.L. (2021). Influence of restorative design on the progression of peri-implant bone loss: A retrospective study. J. Periodontol..

[28] Tang Y., Wang J., Qiu L., Yu H. (2025). Influence of buccal mucosa width/height ratio, emergence profile and buccal bone width on peri-implant tissues: A prospective one-year study. BMC Oral Health.

[29] Maghsoudi P., Valkenburg C., Ter Gunne L.P., van der Weijden F.G.A. (2025). Retrospective Evaluation of Peri-Implant Maintenance in Patients With Implant-Supported Fixed Prostheses. Int. J. Dent..

[30] Lops D., Romeo E., Calza S., Palazzolo A., Viviani L., Salgarello S., Buffoli B., Mensi M. (2022). Association between Peri-Implant Soft Tissue Health and Different Prosthetic Emergence Angles in Esthetic Areas: Digital Evaluation after 3 Years’ Function. J. Clin. Med..

[31] Lops D., Romeo E., Stocchero M., Palazzolo A., Manfredi B., Sbricoli L. (2022). Marginal Bone Maintenance and Different Prosthetic Emergence Angles: A 3-Year Retrospective Study. J. Clin. Med..

[32] Yu X., Li Y., Xie Y., Bi M., Li H., Roccuzzo A., Tonetti M.S. (2025). Emergence angle and profile at implant-supported crowns with and without cantilever extension: A digital study in molar sites. J. Dent..

[33] Siegenthaler M., Strauss F.J., Gamper F., Hämmerle C.H.F., Jung R.E., Thoma D.S. (2022). Anterior implant restorations with a convex emergence profile increase the frequency of recession: 12-month results of a randomized controlled clinical trial. J. Clin. Periodontol..

[34] Endres J., Strauss F.J., Siegenthaler M., Naenni N., Jung R.E., Thoma D.S. (2025). Convex Versus Concave Emergence Profile of Implant-Supported Crowns in the Aesthetic Zone: 3-Year Results of a Randomized Controlled Trial. J. Clin. Periodontol..

